# Rapid *KRAS*, *EGFR*, *BRAF* and *PIK3CA* Mutation Analysis of Fine Needle Aspirates from Non-Small-Cell Lung Cancer Using Allele-Specific qPCR

**DOI:** 10.1371/journal.pone.0017791

**Published:** 2011-03-08

**Authors:** Ronald van Eijk, Jappe Licht, Melanie Schrumpf, Mehrdad Talebian Yazdi, Dina Ruano, Giusi I. Forte, Petra M. Nederlof, Maud Veselic, Klaus F. Rabe, Jouke T. Annema, Vincent Smit, Hans Morreau, Tom van Wezel

**Affiliations:** 1 Department of Pathology, Leiden University Medical Center, Leiden, The Netherlands; 2 Department of Pulmonology, Leiden University Medical Center, Leiden, The Netherlands; 3 Department of Pathology, Netherlands Cancer Institute NKI-AVL, Amsterdam, The Netherlands; University of Texas M. D. Anderson Cancer Center, United States of America

## Abstract

Endobronchial Ultrasound Guided Transbronchial Needle Aspiration (EBUS-TBNA) and Trans-esophageal Ultrasound Scanning with Fine Needle Aspiration (EUS-FNA) are important, novel techniques for the diagnosis and staging of non-small cell lung cancer (NSCLC) that have been incorporated into lung cancer staging guidelines. To guide and optimize treatment decisions, especially for NSCLC patients in stage III and IV, *EGFR* and *KRAS* mutation status is often required. The concordance rate of the mutation analysis between these cytological aspirates and histological samples obtained by surgical staging is unknown. Therefore, we studied the extent to which allele-specific quantitative real-time PCR with hydrolysis probes could be reliably performed on EBUS and EUS fine needle aspirates by comparing the results with histological material from the same patient. We analyzed a series of 43 NSCLC patients for whom cytological and histological material was available. We demonstrated that these standard molecular techniques can be accurately applied on fine needle cytological aspirates from NSCLC patients. Importantly, we show that all mutations detected in the histological material of primary tumor were also identified in the cytological samples. We conclude that molecular profiling can be reliably performed on fine needle cytology aspirates from NSCLC patients.

## Introduction

Lung cancer is the leading cause of cancer mortality in the Western world [1]. For clinical and therapeutic purposes, lung cancer is traditionally subdivided into small cell (SCLC) and non-small cell lung cancer (NSCLC). Whereas SCLC is treated by chemo- and/or radiotherapy, NSCLC is primarily treated through resection; however, only 30% of NSCLC patients have a resectable disease (stage I/II) at the time of presentation [2]. This underscores the importance of accurate, preoperative mediastinal staging in preventing unnecessary resections. Preoperative staging can be performed through the transbronchial (EBUS-TBNA) or transesophageal (EUS-FNA) aspiration of the mediastinal lymph nodes. These cytological procedures are less invasive than routine mediastinoscopy followed by biopsy of the lymph nodes, but similar high specificity and sensitivity [3]–[9] are achieved. Endosonography has been incorporated into lung cancer staging guidelines as an alternative for the surgical staging of the mediastinum [10], [11].

In many cases, the increased use of these minimally invasive techniques is sufficient to diagnose and stage the patient correctly. Although the amount of cellular material obtained by these procedures is relatively small, the information requested by the clinicians is rapidly growing, e.g., for NSCLC, immunohistochemistry and molecular pathology have become part of the standard care [12].

In addition to this change in staging procedures, the rapid development of new medical treatments for NSCLC patients has taken place. A subset of NSCLC cancers may harbor an activating mutation in the EGFR kinase domain [13]. Tumors with these mutations are frequently sensitive to tyrosine kinase inhibitors (TKIs). On the other hand, activating mutations in *KRAS* are associated with resistance to TKIs. Although most publications report that these mutations are mutually exclusive [14]–[19], evidence suggests [20] that a tumor can simultaneously harbor an activating *EGFR* mutation and mutations downstream in the pathway in the *KRAS* gene, which means that upstream inhibition of EGFR will have no therapeutic effect in these cases. Also, mutations in *BRAF* and *PIK3CA* are reported in NSCLC. However, further research is required to determine the extent to which these mutations can have consequences for treatment [21], [22].

Due to these developments and the desire of patients and clinicians to minimize the delay of treatment, rapid and sensitive molecular techniques are needed. Preferably, these techniques should be applicable on formalin-fixed, paraffin-embedded (FFPE) cytological samples [23]–[25] because EBUS-TBNA and EUS-FNA aspiration samples are often the first material that is acquired from patients with NSCLC. Allele-specific quantitative real-time PCR (qPCR) with hydrolysis probes is a reliable and sensitive technique that can be used for this purpose. Detecting mutations in *EGFR*, *KRAS*, *BRAF* and *PIK3CA* with hydrolysis probes has been previously described in NSCLC patients [23]–[26]. The sensitivity of the assays also surpasses the 1% sensitivity proposal set for *KRAS* mutation testing [27].

The majority of *EGFR* mutations are p.L858R, the hotspot mutation in exon 21 and deletions in exon 19, which are reported to comprise up to 36% of all activating mutations [15], [28]. *KRAS* is mutated in 10%–30% of lung carcinomas and over 95% of all activating mutations in *KRAS* are located in exon 1 (codons 12 and 13) [28], [29]. The *BRAF* p.V600E hotspot mutation is reported in 3% of NSCLC and alters residues important in AKT-mediated BRAF phosphorylation, suggesting that the disruption of AKT-induced BRAF inhibition plays a role in malignant transformation [28], [30]. Three hotspot mutations in *PIK3CA* may be another cause of the over-activation of the PI3K–AKT pathway, which promotes the malignant transformation of human airway epithelial cells and has been reported in approximately 4% of lung carcinomas [28], [31].

In the current study, we compared allele-specific qPCR assays for the most frequent activating mutations in *EGFR*, *KRAS*, *BRAF* and *PIK3CA* in tumor-positive fine needle cytological aspirates against histological material of primary tumors.

With this approach, we aimed to determine the extent to which allele-specific qPCR with hydrolysis probes can be performed on cytological aspiration material by comparing the mutation status and then observing the concordance rate between the cytological and histological material and between primary tumors and metastases.

## Materials and Methods

### Ethics Statement

Specific need for ethics committee's approval was not necessary for this study. All samples were handled according to the medical ethical guidelines described in the Code Proper Secondary Use of Human Tissue established by the Dutch Federation of Medical Sciences (www.federa.org, accessed October 27, 2010). Accordingly to these guidelines all human material used in this study has been anonymized since clinical data were not used. Because of this anonymization procedure individual patients' permission is not needed.

### Sample selection

Material from 43 patients with NSCLC for which both tumor-positive cytological and histological material was available were selected from the Department of Pathology in the Leiden University Medical Center (LUMC) and identified through a PALGA database search; non-gynecologic cytological samples between 2005 and 2009 were searched using the search-strings “lung, malignant cells and non small cell lung cancer” and “mediastinum, malignant cells and non small cell lung cancer”. From the 447 unique cytological samples, we selected cases for which tumor-positive histological material of the primary tumor was also available (Supplementary Table S1). Of the 43 patients, 33 patients were subtyped: 14 squamous cell carcinomas, 15 adenocarcinomas, 3 adenosquamous carcinomas and 1 large cell carcinoma. The remaining 10 patients had been classified NSCLC only.

DNA from 42 control FFPE samples was obtained from the Molecular Diagnostics (MD) section of the Department of Pathology in the LUMC. For validation purposes, a series of 10 DNA samples, of which 9 had a demonstrated *EGFR* exon 19 deletion by DNA sequencing, was provided by the Netherlands Cancer Institute - Antoni van Leeuwenhoek Hospital.

### DNA isolation

**Figure 1:**
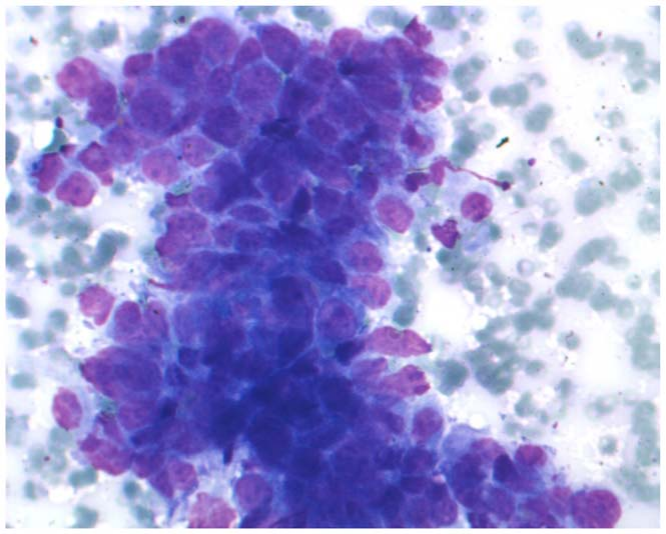
Mediastinal lymph node cytology of a NSCLC patient. Microscopical detail of a cytological smear obtained through fine needle aspiration of a meadiastinal lymph node from a NSCLC patient. The tumor foci are marked on the backside of each slide with a diamond tip. Subsequently the coverslips are removed and tumor foci are scraped from the slide using a scalpel blade (not shown).

Prior to DNA isolation, tumor cells were enriched to obtain tumor cell percentages >70% (Figure 1). The FFPE tumor blocks were enriched for tumor cells guided by a hematoxylin and eosin (H&E)-stained slide taking 0.6-mm tissue punches from the tumor focus in the FFPE block by using a tissue microarrayer (Beecher Instruments, Sun Prairie, WI, USA). Prior to DNA isolation, the tissue was deparaffinized in xylene and washed in 70% ethanol. For the cell blocks, 10 slides of 10 µm were stained with hematoxylin. Tumor cells were marked by guiding with a 5-µm H&E slide and the corresponding tumor fields on the hematoxylin slides were microdissected.

For the cytology smears, microdissection was initiated by marking the tumor foci with a diamond needle on the back side of the Giemsa-stained slide. Subsequently, cover slips were removed by incubating in xylene at room temperature in separate 50-ml tubes to avoid contamination. Incubation was performed overnight or until the cover slip was removed (sometimes up to a week). Subsequently, the slides were washed in alcohol, three times in 100%, once in 70% and once in 50%, to rehydrate the tissue. Using a scalpel blade, the tumor foci from the marked areas were scraped and collected in micro tubes for DNA isolation.

DNA was isolated using the NucleoSpin Tissue XS Genomic DNA Purification kit (Machery-Nagel, Düren, Germany) according to the manufacturer's instructions. The average DNA yield from the cytological smears and cell blocks was 282 ng and 280 ng, respectively. However, cytology smears were fixed using methanol rather than formalin, so the isolated DNA was expected to be of higher quality. The average DNA yield from the biopsies was considerably higher (985 ng).

Prior to analysis, the DNA samples were diluted by 5 or 15 times. We observed that DNA diluted over 15 times generally gave a quantification cycle (Cq)>35 (data not shown); therefore, in the subsequent assays, we used 5× stock DNA dilutions in sterile water.

### Mutation detection

The assays for the detection of seven different *KRAS*, three *PIK3CA* and one *BRAF* variant were obtained through the Custom TaqMan® Assay Design Tool (Applied Biosystems, Nieuwerkerk a/d IJssel, NL). Hydrolysis probes were designed with minor grove binder (MGB) modifications at the 3′-end. These modified probes have the advantage that relatively short probes can be designed with higher melting temperature (Tm) and increased duplex stability and specificity in comparison to conventional probes [32]. The *EGFR* assays were described previously [33]. qPCR reactions were performed in 10-µl reactions containing 5 µl of FastStart Universal Probe Master (Roche Applied Science), 1 µl of 10× primer and hydrolysis probe solutions, 2 µl of 5× diluted DNA and 2 µl of sterile water in a sealed LightCycler 480 Multiwell Plate 384 (Roche Applied Science) in a LightCycler 480 system (Roche Diagnostics) as follows: 10 minutes at 95°C and 45 cycles of 15 seconds at 92°C, 60 seconds at 60°C and 10 seconds at 72°C. For validation, we performed direct Sanger sequencing using M13 primers as described previously [34] at the sequencing core of the Leiden Genome Technology Center. Primer sequences are listed in Supplementary Table S2. All DNA Sequencing was completed on known genes and no new sequencing was completed.

**Figure 2:**
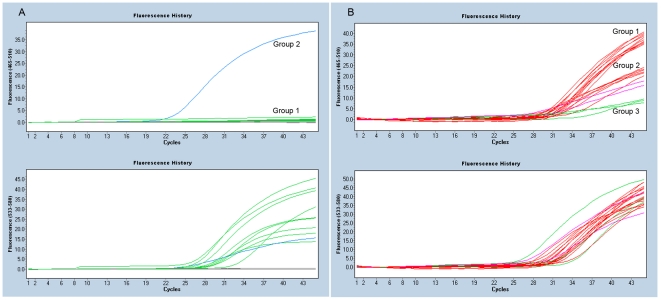
qPCR Results for the EGFR assays. Panel A shows the EGFR p.L858R assay. All samples show a wild type (control) signal, VIC, lower panel (green and blue lines) while only group 2 (blue line) shows a mutant FAM signal. Panel B shows the EGFR exon 19 mutation assay. The lower panel shows the wildtype VIC signal for all samples (red, green and purple lines). The top panels shows the mutant FAM signal. Group 1 (red lines) shows the wildtype signal, Group 2 (red and purple) shows possible mutants with decreased fluorescence, group 3 (green line) show an almost completely disappeared signal indicating a deletion. The images are obtained from the LC480 software release 1.5.0. The y-axis shows the relative fluorescence for the FAM (465–510 nm) and VIC (533–580nm) probes, x-axis shows the PCR cycles.

Raw data from the LC480 software were imported into an in-house–created Microsoft Excel 2003 spreadsheet to define the mutation status. The quantification cycle (Cq) was used for quality assessment and samples with Cq values exceeding 35 (Cq>35) in the wild-type channel were rejected and excluded for further analysis. To determine the presence or absence of a mutation, the endpoint fluorescence ratio R_m_/R_wt_ was calculated after subtracting the average background signal from three negative controls. The spreadsheet is available upon request. For *BRAF*, *PIK3CA* and *EGFR* p.L858R, mutation status was directly discriminated (Figure 2a). Mutations were identified when the R_m_/R_wt_ ratio was higher than 0.7, while a ratio lower than 0.3 indicated the absence of a mutation. No intermediate values were observed. In *KRAS* wild-type samples, an increased background signal was observed for the c.34G>T (R_m_/R_wt_±0.4) and c.38G>C (R_m_/R_wt_±0.6) assay in the mutant probe channel. This was probably caused by imperfect hybridization of these probes to the wild-type allele. The setting to identify the mutation correctly was c.34G>T R_m_/R_wt_>0.7, while the c.38G>T mutant was identified when an R_m_/R_wt_ ratio cut-off of 0.8 was used. The *EGFR* exon 19 deletion probe resulted in a drop in endpoint fluorescence, while in a wild-type sample, both probes gave a signal. To analyze *EGFR* exon 19 deletions, R_m_/R_wt_>0.8 and Cq<32 were considered wild-type and R_m_/R_wt_≤0.6 and Cq<32 indicated a deletion. Intermediate values, with R_m_/R_wt_ ratio between 0.6 and 0.8 and Cq<35, required confirmation using Sanger sequencing.

## Results and Discussion

### Assay design and validation

For *BRAF*, a single assay was designed that detects the activating hotspot mutation p.V600E, which results from the c.1799T>A substitution [35]. For *PIK3CA*, we designed probes for the three most common substitutions [36]: c.1624G>A (p.E542K), c.1633G>A (p.E545K) and c.3140A>G (p.H1047R). Although these three assays detected over 85% of all mutations in NSCLC, some of the infrequent substitutions in the hotspot regions were potentially missed. For *KRAS*, we designed assays for the seven most frequent base pair substitutions in codons 12 and 13: c.34G>A (p.G12S), c.34G>C, (p.G12R), c.34G>T, (p.G12C), c.35G>A, (p.G12D), c.35G>C, (p.G12A), c.35G>T (p.G12V) and c.38G>A (p.G13D). Together, these assays detect almost all substitutions in *KRAS*, although some rare variants might be missed. To detect the p.L858R hotspot and exon 19 deletions in *EGFR* we used previously reported assays [33]. The *EGFR* p.L858R mutation was detected using a probe mix containing a wild-type probe and two different mutant probes: one for the most common variant (c.2573T>G) and one for the rare complex c.2573_2574TG>GT inversion.

### Hotspot mutation analysis in cytology material from NSCLC patients

To address the extent to which the mutation analysis can be reliably performed on EBUS-TBNA and EUS-FNA aspiration material, we performed the 13 assays on 43 patients with NSCLC for which both primary tumor (either biopsies or histological material from resections) and tumor-positive cytological material had been collected. The material from the 43 patients represents 29 tissue cores from histological excisions, 23 microdissected biopsies and 3 whole-section biopsies which were compared to 45 microdissected cytological smears and 17 microdissected cell blocks (Supplementary Table S1).

Six patients presented with a *KRAS* mutation: c.34G>T (N = 2), c.34G>A, c.35G>A, c.35G>C and c.38G>A. One patient carried a deletion in exon 19 of *EGFR* (c.2238_2252del15) and two patients showed *PIK3CA* mutations: c.1633G>A and c.3140A>G. The latter case showed an additional *KRAS* mutation (p.34G>T). No mutations in *BRAF* were observed.

For some patients, multiple histological and/or cytological samples were analyzed. In different samples for the same patient, conflicting results for the same type of material were never observed. Therefore in table 1 each patient is represented only once, where for each type of material the information from all the patient's samples is merged. This means that the clearest signal for each assay took precedence. In table 1, the remaining missing calls, due to low signals are indicated by “?”.

**Table 1:** Results of mutation analysis for the 13 assays for 43 NSCLC subjects.

	*KRAS*							*EGFR*		*BRAF*	*PIK3CA*		
SAMPLE	p.G12S	p.G12R	p.G12C	p.G12D	p.G12A	p.G12V	p.G13D	deletion	p.L858R	p.V600E	p.E542K	p.E545K	p.H1047R
ORIGIN	c.34G>A	c.34G>C	c.34G>T	c.35G>A	c.35G>C	c.35G>T	c.38G>A	exon 19	exon 21	c.1799T>A	c.1624G>A	c.1633G>A	c.3140A>G
1-M	√	√	√	√	√	√	√	√	√	√	√	√	√
2-M	√	√	√	mut	√	√	√	√	√	√	√	√	√
3-P	√	√	√	√	−/?	√	−/?	−/?	√	−/?	−/?	−/?	−/?
4-M	√	√	√	√	√	√	√	√	√	√	√	√	√
5-M	√	√	√	√	√	√	√	√	√	?/−	?/−	√	?/−
6-M	?/−	√	√	√	√	√	√	√	√	√	√	√	√
7-M	√	√	√	√	√	√	√	√	√	√	√	√	√
8-M	√	√	√	√	√	√	√	√	√	√	√	√	√
9-M	√	√	√	√	√	√	√	√	√	√	−/?	−/?	−/?
10-M	√	√	√	√	√	√	√	√	√	√	√	√	√
11-M	√	√	√	−/?	−/?	√	√	√	√	√	√	√	√
12-M	√	√	√	√	√	√	√	√	√	√	√	√	√
13-M	√	√	√	√	√	√	√	√	√	√	√	√	√
14-M	√	√	√	√	√	√	√	√	√	√	√	√	√
15-M	√	√	√	√	√	√	√	√	√	√	√	√	√
16-M	√	√	√	√	√	√	√	√	√	√	√	√	√
17-M	√	√	√	√	√	√	√	√	√	√	−/?	−/?	−/?
18-M	√	√	√	√	√	√	√	√	√	√	√	√	√
19-P	√	√	√	√	√	√	√	mut	√	√	√	√	√
20-P	√	√	√	√	√	√	√	√	√	√	√	√	√
21-P	−/?	−/?	−/?	−/?	−/?	−/?	√	−/?	√	−/?	−/?	−/?	−/?
22-P	√	√	√	√	√	√	√	√	√	√	√	√	√
23-P	√	√	√	√	√	√	√	√	−/?	√	−/?	−/?	√
24-P	√	√	√	√	√	√	√	√	−/?	√	−/?	−/?	−/?
25-P	√	√	√	√	√	√	√	?/−	√	?/−	?/−	√	?/−
26-P	√	√	mut	√	√	√	√	√	√	√	√	√	√
27-P	√	√	√	√	√	√	√	√	√	√	√	√	√
28-P	√	√	√	√	√	√	√	−/?	√	√	−/?	−/?	−/?
29-P	√	√	√	√	√	√	mut	√	√	√	√	√	√
30-P	√	√	√	√	√	√	√	√	√	√	√	mut	√
31-M	√	√	√	√	√	√	√	√	√	√	√	√	√
32-M	√	√	mut	√	√	√	√	√	√	√	√	√	√
33-M	√	√	√	√	√	√	√	√	√	√	√	√	√
34-M	√	√	√	√	√	√	√	√	√	√	√	√	√
35-P	√	√	mut	−/?	−/?	−/?	√	√	√	√	√	√	mut
36-M	√	√	√	√	√	√	√	√	√	√	√	√	√
37-M	√	−/?	√	−/?	√	√	−/?	−/?	√	−/?	√	√	√
38-P	√	√	√	√	√	√	√	√	√	√	√	√	√
39-M	√	√	mut	√	√	√	√	√	√	√	√	√	√
40-M	−/+	√	√	√	√	√	√	√	√	√	√	√	√
41-M	√	√	√	√	√	√	√	√	√	√	−/?	−/?	−/?
42-M	√	√	√	√	√	√	√	√	√	√	√	√	√
43-M	√	√	√	√	√	√	√	√	√	√	√	√	√

For each subject the origin of the cytological material and the mutational status for each of the 13 assays are indicated. Only one subject (40) shows a discordance on a single assay (boxed cell), which may be explained from the commonly observed genetic divergence of metastasis from its primary tumor.

**‘P’**, primary tumor; **‘M’**, metastasis; **‘√’**, concordance of wildtype result from mutation analysis between histology and cytology; **‘mut’**, concordant samples with a somatic mutation; ‘**−/+**’, discordant result, mutation in the cytological and wildtype in the histological material; ‘**−/?**’, wildtype signal in the histology with discordancy in the cytological material because of low signal; ‘**?/−**’, Low signal in the histological material and wildtype signal in the cytology.

The overall call rate in the 13 assays, after merging, amounts to 95% (58 undetermined results out of 1118 tests). The call rate for histological material is substantially higher at 99% (8 undetermined out of 559) than for cytological material at 91% (48 out of 559). Within the cytological material, the call rate for primary tumors is lower (84%) than for metastases (96%). Note that these observations remain if the patient with the lowest quality results (sample 21) is removed. Within the histological material the same difference in call rate can be observed, but in a much lower degree (98% for primary tumors versus 99% for metastases). When comparing call rates per assay, we observed that the three assays on the *PIK3CA* gene performed less (between 88 and 91%) than the other 10 assays (between 94 and 99%).

As could be observed, when cytological material was obtained from primary tumors, the mutation results for histology and cytology were concordant in all cases where both results were determined. When cytological material was obtained from metastasis, in one patient (nr 40) with an adenocarcinoma/bronchoalveolar cell carcinoma (BAC), a KRAS c.34G>A mutation was identified in the mediastinal lymph node which was not detected in the primary tumor. This could be explained by the commonly observed genetic divergence of metastasis from its primary tumor. In this case the time-span is 18 years between the primary tumor and the metastasis. Overall, the discordance rate is only 0.20% (1 assay out of 503 where both histological and cytological results are determined).

### Tumor cell percentage and DNA quality

From biopsies and cytology, only small tumor foci can be microdissected. This results in a low DNA yield that, in case of formalin fixation, is also partially degraded. To study the quality of the DNA, we compared the DNA yield to assay performance. We observed that the average amount of DNA isolated (295 ng) was lower in the group (n = 15) where two or more assays failed [as compared to the group without failing assays (n = 102, 2973 ng)]. Nevertheless, in the latter group, 44% of the samples (n = 45) also had a DNA amount of lower than 295 ng. This indicates that Cq values are a better indicator of DNA quality and performance than DNA concentration measurements.

**Figure 3:**
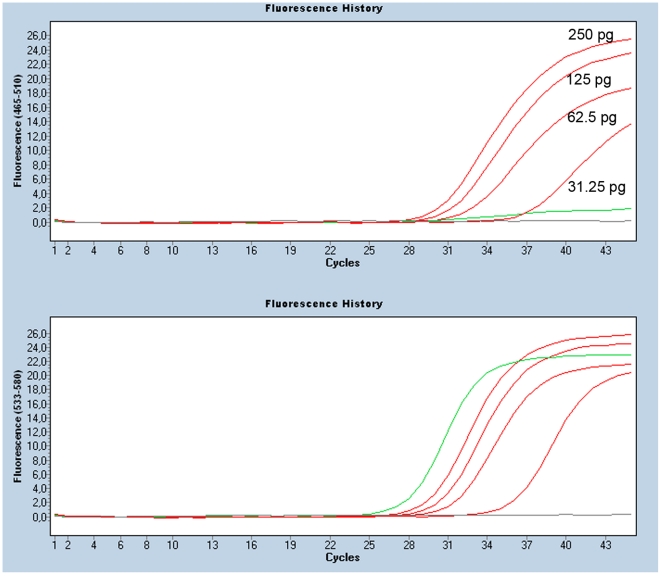
Effect of the DNA concentration on the c.34G>T KRAS assay. The top panel shows the mutant (FAM) signal for a range of different amounts of input DNA in pg carrying the c.34G>T KRAS mutation. No “mutant” signal is observed in a wildtype DNA (green line) and water control (grey line). In the wildtype (VIC) panel all DNA's show a wildtype signal while the water control is negative (grey line). The images are obtained from the LC480 software release 1.5.0. The y-axis shows the relative fluorescence for the FAM (465–510 nm) and VIC (533–580nm) probes, x-axis shows the PCR cycles.

Allele-specific qPCR with hydrolysis probes has been reported to surpass the 1% sensitivity level [27]. However, considering that the qPCR efficiency also depends on DNA fragmentation, the DNA isolated from FFPE samples could accurately be analyzed at a sensitivity level of 10% [26]. We determined the detection limit in serial dilutions of DNA from two tumors carrying a *KRAS* c.34G>T or a c.35G>A mutation. This showed that the minimal DNA input must be at least 32 pg, the equivalent of 4–6 cells of high molecular DNA, to give Cq values <35 (Figure 3).

Furthermore, we validated the assays in a series of DNA isolates from microdissected FFPE samples with known *KRAS*, *PIK3CA*, *BRAF* or EFGR variants as determined by Sanger sequencing. We found a 100% correlation with the hydrolysis probe assays.

We validated the assay for *EGFR* exon 19 in a series of 10 samples with possible sequence verified exon 19 deletions and a tumor percentage of more than 50%. The samples were tested without prior knowledge of the mutation status. The hydrolysis assay results were compared with the DNA sequence results and all nine samples containing an exon 19 deletion were correctly identified and distinguished from the wild-type specimen (Figure 2b). In one case, there was an 18-bp insertion in exon 19. Because this fell outside the detection area of the probes, the mutant was not detected by the deletion assay (SampleID 1012 in Supplementary Table S3). These results show that all hotspot mutations and *EGFR* exon 19 deletions can be detected using the hydrolysis probes.

### Cross-reactivity

Mutations in *KRAS* and *PIK3CA* cluster in hotspots. For *KRAS*, all seven assays hybridized to codons 12 and 13 (nucleotides c.34G, c.35G and c.38G), while for *PIK3CA* two assays detected exon 9 changes (c.1624G>A and c.1633G>A). As the probes potentially hybridized in the same region, cross-reactivity between the different *KRAS* or *PIK3CA* assays might be observed as a result of increased fluorescence readings from imperfectly matched probes or primers [26]. Additionally, cross-reactivity might result from (rare) base pair substitutions that are not covered by the used assays.

Cross-reactivity was studied in a series of 42 MD samples carrying a *KRAS* mutation at position 34, 35 or 38. A total of 294 assays (42×7) were performed. The correct mutation status was identified when an R_m_/R_wt_ ratio cut-off >0.7 was used; however, in 68 assays, a cross-reactivity signal was observed. Five cross-reactivity signals had R_m_/R_wt_>0.7, but in these cases, the assay for the genuine mutation had R_m_/R_wt_>1.0. Cross-reactivity was only observed for probes covering the same base pair position (at position 34 or 35). Cross-reactivity between signals from base pair 34 or 35 and position 38 was not observed (Supplementary Table S4). Therefore, it is probable that no cross-reactivity effects were observed for the two different *PIK3CA* probes.

### Clinical practice

The described methods can be implemented in clinical practice. The molecular diagnostics test results can be generated in a short time. In daily practice, the cytological EBUS-TBNA or EUS-FNA aspiration material is morphologically typed by pathologists. Subsequently, samples are clustered for microdissection on a weekly basis. Microdissection is essential to obtain high tumor cell percentages to detect the EGFR exon 19 deletion, and to allow other analyses with lower sensitivity than the described method, e.g Sanger sequencing. After DNA isolation, the hydrolysis probe assays are performed on the DNA dilutions. At the end of the second day, the qPCR results are analyzed in an in-house–developed Microsoft Excel–based analysis tool to interpret the results, e.g., determine the mutation status of each probe and interpret the effect of cross-reactivity. The results are subsequently reported to the clinic. A limitation of hotspot analysis is, by definition, that only the hotspot mutations are detected, while Sanger sequencing can identify all mutations in the PCR amplicon. In some cases, in which the mutation analysis does not meet the quality settings, Sanger sequencing will be performed. For Sanger sequencing, extra PCR reactions, reaction product purifications and electrophoresis must be performed, which will require two extra days in the analysis pipeline.

### Conclusion

We conclude that somatic mutation hotspot analysis for *KRAS*, *PIK3CA*, *BRAF* and *EGFR* of fine needle aspirations of mediastinal lymph nodes in NSCLC patients is accurate and reliable. Somatic hotspot mutation analysis for *KRAS*, *PIK3CA*, *BRAF* and *EGFR* can reliably be performed using allele-specific qPCR with hydrolysis probes; the mutation results from cytological specimens and the primary tumors are highly concordant.

Somatic mutation analysis in NSCLC for molecular staging and the guidance of treatment decisions can be performed on EBUS and EUS fine needle aspirates, procedures that are less invasive for the patient than routine mediastinoscopy.

Our findings indicate that the molecular genetic analysis of NSCLC should be incorporated with the standard EBUS and EUS procedures. This combined approach will result in the accurate diagnosing and staging of those patients and will also help to guide the optimal treatment decisions, especially in stage III and IV NSCLC.

## Supporting Information

Table S1 General overview.
(XLS)

Table S2 Primer sequences.
(XLS)

Table S3 EGFR exon 19 validation experiment.
(XLS)

Table S4 Cross reactivity in KRAS assays.
(XLS)

## References

[1] Parkin DM, Bray F, Ferlay J, Pisani P (2005). Global Cancer Statistics, 2002.. CA Cancer J Clin.

[2] Molina JR, Adjei AA, Jett JR (2006). Advances in Chemotherapy of Non-small Cell Lung Cancer*.. Chest.

[3] Gu P, Zhao YZ, Jiang LY, Zhang W, Xin Y (2009). Endobronchial ultrasound-guided transbronchial needle aspiration for staging of lung cancer: a systematic review and meta-analysis.. Eur J Cancer.

[4] Annema JT, Versteegh MI, Veselic M, Welker L, Mauad T (2005). Endoscopic Ultrasound Added to Mediastinoscopy for Preoperative Staging of Patients With Lung Cancer.. JAMA.

[5] Horiike A, Kimura H, Nishio K, Ohyanagi F, Satoh Y (2007). Detection of Epidermal Growth Factor Receptor Mutation in Transbronchial Needle Aspirates of Non-Small Cell Lung Cancer*.. Chest.

[6] Nakajima T, Yasufuku K, Suzuki M, Hiroshima K, Kubo R (2007). Assessment of Epidermal Growth Factor Receptor Mutation by Endobronchial Ultrasound-Guided Transbronchial Needle Aspiration*.. Chest.

[7] Tournoy KG, Rintoul RC, van Meerbeeck JP, Carroll NR, Praet M (2009). EBUS-TBNA for the diagnosis of central parenchymal lung lesions not visible at routine bronchoscopy.. Lung Cancer.

[8] Micames CG, McCrory DC, Pavey DA, Jowell PS, Gress FG (2007). Endoscopic ultrasound-guided fine-needle aspiration for non-small cell lung cancer staging: A systematic review and metaanalysis.. Chest.

[9] Annema JT, Versteegh MI, Veselic M, Voigt P, Rabe KF (2005). Endoscopic ultrasound-guided fine-needle aspiration in the diagnosis and staging of lung cancer and its impact on surgical staging.. J Clin Oncol.

[10] Detterbeck FC, Jantz MA, Wallace M, Vansteenkiste J, Silvestri GA (2007). Invasive mediastinal staging of lung cancer: ACCP evidence-based clinical practice guidelines (2nd edition).. Chest.

[11] De Leyn P, Lardinois D, Van Schil PE, Rami-Porta R, Passlick B (2007). ESTS guidelines for preoperative lymph node staging for non-small cell lung cancer.. Eur J Cardiothorac Surg.

[12] Rossi G, Pelosi G, Graziano P, Barbareschi M, Papotti M (2009). Review Article: A Reevaluation of the Clinical Significance of Histological Subtyping of Non–Small-Cell Lung Carcinoma: Diagnostic Algorithms in the Era of Personalized Treatments.. International Journal of Surgical Pathology.

[13] Gazdar AF (2009). Activating and resistance mutations of EGFR in non-small-cell lung cancer: role in clinical response to EGFR tyrosine kinase inhibitors.. Oncogene.

[14] Jang TW, Oak CH, Chang HK, Suo SJ, Jung MH (2009). EGFR and KRAS mutations in patients with adenocarcinoma of the lung.. Korean J Intern Med.

[15] Ladanyi M, Pao W (2008). Lung adenocarcinoma: guiding EGFR-targeted therapy and beyond.. Mod Pathol.

[16] Lim EH, Zhang SL, Li JL, Yap WS, Howe TC (2009). Using whole genome amplification (WGA) of low-volume biopsies to assess the prognostic role of EGFR, KRAS, p53, and CMET mutations in advanced-stage non-small cell lung cancer (NSCLC).. J Thorac Oncol.

[17] Monaco SE, Nikiforova MN, Cieply K, Teot LA, Khalbuss WE (2010). A comparison of EGFR and KRAS status in primary lung carcinoma and matched metastases.. Human Pathology.

[18] Motoi N, Szoke J, Riely GJ, Seshan VE, Kris MG (2008). Lung Adenocarcinoma: Modification of the 2004 WHO Mixed Subtype to Include the Major Histologic Subtype Suggests Correlations Between Papillary and Micropapillary Adenocarcinoma Subtypes, EGFR Mutations and Gene Expression Analysis.. The American Journal of Surgical Pathology.

[19] Sasaki H, Okuda K, Kawano O, Endo K, Yukiue H (2007). Nras and Kras mutation in Japanese lung cancer patients: Genotyping analysis using LightCycler.. Oncol Rep.

[20] Kalikaki A, Koutsopoulos A, Trypaki M, Souglakos J, Stathopoulos E (2008). Comparison of EGFR and K-RAS gene status between primary tumours and corresponding metastases in NSCLC.. Br J Cancer.

[21] Hirsch FR, Varella-Garcia M, Bunn PA, Franklin WA, Dziadziuszko R (2006). Molecular Predictors of Outcome With Gefitinib in a Phase III Placebo-Controlled Study in Advanced Non-Small-Cell Lung Cancer.. J Clin Oncol.

[22] Zou ZQ, Zhang XH, Wang F, Shen QJ, Xu J (2009). A novel dual PI3Kalpha/mTOR inhibitor PI-103 with high antitumor activity in non-small cell lung cancer cells.. Int J Mol Med.

[23] Boldrini L, Gisfredi S, Ursino S, Camacci T, Baldini E (2007). Mutational Analysis in Cytological Specimens of Advanced Lung Adenocarcinoma: A Sensitive Method for Molecular Diagnosis.. Journal of Thoracic Oncology.

[24] Endo K, Konishi A, Sasaki H, Takada M, Tanaka H (2005). Epidermal growth factor receptor gene mutation in non-small cell lung cancer using highly sensitive and fast TaqMan PCR assay.. Lung Cancer.

[25] Molina-Vila MA, Bertran-Alamillo J, Reguart N, Taron M, CastellÃ E (2008). A Sensitive Method for Detecting EGFR Mutations in Non-small Cell Lung Cancer Samples with Few Tumor Cells.. Journal of Thoracic Oncology.

[26] Kotoula V, Charalambous E, Biesmans B, Malousi A, Vrettou E (2009). Targeted KRAS mutation assessment on patient tumor histologic material in real time diagnostics.. PLoS One.

[27] van Krieken JH, Jung A, Kirchner T, Carneiro F, Seruca R (2008). KRAS mutation testing for predicting response to anti-EGFR therapy for colorectal carcinoma: proposal for an European quality assurance program.. Virchows Arch.

[28] Forbes SA, Bhamra G, Bamford S, Dawson E, Kok C (2008). The Catalogue of Somatic Mutations in Cancer (COSMIC).. Curr Protoc Hum Genet Chapter.

[29] Jancik S, Drabek J, Radzioch D, Hajduch M (2010). Clinical relevance of KRAS in human cancers.. J Biomed Biotechnol.

[30] Brose MS, Volpe P, Feldman M, Kumar M, Rishi I (2002). BRAF and RAS Mutations in Human Lung Cancer and Melanoma.. Cancer Research.

[31] Okudela K, Suzuki M, Kageyama S, Bunai T, Nagura K (2007). PIK3CA mutation and amplification in human lung cancer.. Pathol Int.

[32] Kutyavin IV, Afonina IA, Mills A, Gorn VV, Lukhtanov EA (2000). 3′-minor groove binder-DNA probes increase sequence specificity at PCR extension temperatures.. Nucleic Acids Res.

[33] Yung TK, Chan KC, Mok TS, Tong J, To KF (2009). Single-molecule detection of epidermal growth factor receptor mutations in plasma by microfluidics digital PCR in non-small cell lung cancer patients.. Clin Cancer Res.

[34] van Eijk R, van Puijenbroek M, Chhatta AR, Gupta N, Vossen RH (2010). Sensitive and specific KRAS somatic mutation analysis on whole-genome amplified DNA from archival tissues.. J Mol Diagn.

[35] Benlloch S, Paya A, Alenda C, Bessa X, Andreu M (2006). Detection of BRAF V600E mutation in colorectal cancer: comparison of automatic sequencing and real-time chemistry methodology.. J Mol Diagn.

[36] Samuels Y, Velculescu VE (2004). Oncogenic mutations of PIK3CA in human cancers.. Cell Cycle.

